# Socioeconomic Status, Palliative Care, and Death at Home Among Patients With Cancer Before and During COVID-19

**DOI:** 10.1001/jamanetworkopen.2024.0503

**Published:** 2024-02-27

**Authors:** Javaid Iqbal, Rahim Moineddin, Robert A. Fowler, Monika K. Krzyzanowska, Christopher M. Booth, James Downar, Jenny Lau, Lisa W. Le, Gary Rodin, Hsien Seow, Peter Tanuseputro, Craig C. Earle, Kieran L. Quinn, Breffni Hannon, Camilla Zimmermann

**Affiliations:** 1 Department of Supportive Care, Princess Margaret Cancer Centre, Toronto, Ontario, Canada; 2 Department of Family and Community Medicine, University of Toronto, Toronto, Ontario, Canada; 3 Interdepartmental Division of Critical Care Medicine, University of Toronto, Toronto, Ontario, Canada; 4 Department of Medicine, University of Toronto, Ontario, Canada; 5 Division of Medical Oncology and Hematology, Princess Margaret Cancer Centre, Toronto, Ontario, Canada; 6 Department of Oncology, Queen’s University, Kingston, Ontario, Canada; 7 Division of Palliative Care, Department of Medicine, University of Ottawa, Ottawa, Ontario, Canada; 8 Bruyere Research Institute, Bruyere Continuing Care, Ottawa, Ontario, Canada; 9 Department of Biostatistics, University Health Network, Toronto, Toronto, Ontario, Canada; 10 Department of Psychiatry, Faculty of Medicine, University of Toronto, Toronto, Ontario, Canada; 11 Department of Oncology, McMaster University, Hamilton, Ontario, Canada; 12 Department of Medicine, University of Ottawa, Ottawa, Ontario, Canada

## Abstract

**Importance:**

The COVID-19 pandemic had a profound impact on the delivery of cancer care, but less is known about its association with place of death and delivery of specialized palliative care (SPC) and potential disparities in these outcomes.

**Objective:**

To evaluate the association of the COVID-19 pandemic with death at home and SPC delivery at the end of life and to examine whether disparities in socioeconomic status exist for these outcomes.

**Design, Setting, and Participants:**

In this cohort study, an interrupted time series analysis was conducted using Ontario Cancer Registry data comprising adult patients aged 18 years or older who died with cancer between the pre–COVID-19 (March 16, 2015, to March 15, 2020) and COVID-19 (March 16, 2020, to March 15, 2021) periods. The data analysis was performed between March and November 2023.

**Exposure:**

COVID-19–related hospital restrictions starting March 16, 2020.

**Main Outcomes and Measures:**

Outcomes were death at home and SPC delivery at the end of life (last 30 days before death). Socioeconomic status was measured using Ontario Marginalization Index area-based material deprivation quintiles, with quintile 1 (Q1) indicating the least deprivation; Q3, intermediate deprivation; and Q5, the most deprivation. Segmented linear regression was used to estimate monthly trends in outcomes before, at the start of, and in the first year of the COVID-19 pandemic.

**Results:**

Of 173 915 patients in the study cohort (mean [SD] age, 72.1 [12.5] years; males, 54.1% [95% CI, 53.8%-54.3%]), 83.7% (95% CI, 83.6%-83.9%) died in the pre–COVID-19 period and 16.3% (95% CI, 16.1%-16.4%) died in the COVID-19 period, 54.5% (95% CI, 54.2%-54.7%) died at home during the entire study period, and 57.8% (95% CI, 57.5%-58.0%) received SPC at the end of life. In March 2020, home deaths increased by 8.3% (95% CI, 7.4%-9.1%); however, this increase was less marked in Q5 (6.1%; 95% CI, 4.4%-7.8%) than in Q1 (11.4%; 95% CI, 9.6%-13.2%) and Q3 (10.0%; 95% CI, 9.0%-11.1%). There was a simultaneous decrease of 5.3% (95% CI, −6.3% to –4.4%) in the rate of SPC at the end of life, with no significant difference among quintiles. Patients who received SPC at the end of life (vs no SPC) were more likely to die at home before and during the pandemic. However, there was a larger immediate increase in home deaths among those who received no SPC at the end of life vs those who received SPC (Q1, 17.5% [95% CI, 15.2%-19.8%] vs 7.6% [95% CI, 5.4%-9.7%]; Q3, 12.7% [95% CI, 10.8%-14.5%] vs 9.0% [95% CI, 7.2%-10.7%]). For Q5, the increase in home deaths was significant only for patients who did not receive SPC (13.9% [95% CI, 11.9%-15.8%] vs 1.2% [95% CI, −1.0% to 3.5%]).

**Conclusions and Relevance:**

These findings suggest that the COVID-19 pandemic was associated with amplified socioeconomic disparities in death at home and SPC delivery at the end of life. Future research should focus on the mechanisms of these disparities and on developing interventions to ensure equitable and consistent SPC access.

## Key Points

**Question:**

Was the COVID-19 pandemic associated with increased socioeconomic disparities in the use of specialized palliative care (SPC) and deaths at home among patients with cancer?

**Findings:**

In this cohort study of 173 915 adult patients who died with cancer, the COVID-19 pandemic was associated with a significant immediate increase in home deaths but a decrease in delivery of SPC at the end of life. For patients with low socioeconomic status, the increase in home deaths was smaller and only observed among patients who did not receive SPC.

**Meaning:**

These findings suggest that the COVID-19 pandemic amplified end-of-life care disparities associated with socioeconomic status; future interventions are needed to ensure equitable, consistent access to SPC.

## Teaser

This cohort study evaluated the association of the COVID-19 pandemic with delivery of specialized palliative care and death at home for patients with cancer by socioeconomic status.

## Introduction

Place of death is an important determinant of the end-of-life experience of patients with cancer and their caregivers and is often used as a metric to assess the quality of end-of-life cancer care.^1,2,3^ Prior to the COVID-19 pandemic, studies suggested that most patients with advanced cancer preferred to receive end-of-life care at home and to die at home.^4,5^ Home death was associated with improved patient quality of life at the end of life^1,6,7^ and with better quality of death^2,8^; in turn, delivery of specialized palliative care (SPC) may increase the likelihood of dying at home.^9^ The COVID-19 pandemic had a profound impact on the delivery of cancer care,^10,11^ but less is known about its association with place of death, the delivery of SPC, and potential disparities in these outcomes.

Socioeconomic status (SES) is a contributor to disparities in health care utilization and place of death,^12,13,14,15^ but there has been scant cancer research about the influence of the COVID-19 pandemic in this regard. Area-level material deprivation has been used as a comprehensive composite measure of SES because it accounts not only for individual-level factors, such as income, employment, and education, but also for social factors, such as family structure and housing quality, and the ability of individuals to access and attain basic material needs.^16,17^ A study in England, Wales, Scotland, and Northern Ireland found that there was an increase in home deaths at the beginning of the pandemic, which was lowest for people living in areas with the greatest deprivation.^18^ However, the study did not specifically examine patients with cancer; nor did it evaluate the delivery of SPC or compare monthly trends in home deaths before and after the pandemic.

We addressed these knowledge gaps by quantifying immediate and delayed associations of the COVID-19 pandemic with existing trends in home deaths and delivery of SPC among patients who died with cancer. We stratified these analyses according to SES, measured by area-based material deprivation status, to examine potential disparities in these outcomes.

## Methods

### Study Design and Data Source

We performed a population-based, retrospective cohort study in Ontario, Canada, linking the Ontario Cancer Registry, Discharge Abstract Database, National Ambulatory Care Reporting System, Continuing Care Reporting System, Ontario Health Insurance Plan, and Ontario Marginalization Index databases at ICES (formerly the Institute of Clinical Evaluative Sciences) (eTable 1 in Supplement 1). Ontario is Canada’s most populous province with more than 13 million adults and more than 1.6 million confirmed cases of COVID-19 infection.^19^ Residents of Ontario have public insurance for hospital care and physicians’ services, and those aged 65 years or older are provided prescription drug insurance.

All personal identifiers were removed to protect the privacy of patients, and data linkage was performed using encrypted codes. The University Health Network research ethics board approved the research protocol and waived informed consent. Under section 45 of the Personal Health Information Protection Act, ICES can receive and use health information without consent to analyze and compile statistical information about the Canadian health care system. We followed the Strengthening the Reporting of Observational Studies in Epidemiology (STROBE) reporting guideline for cohort studies (eTable 2 in Supplement 1).^20^

### Study Participants

The study population included all adults aged 18 years or older who died in Ontario between March 16, 2015, and March 15, 2021, and were diagnosed with any cancer (except nonmelanoma skin cancer) within 5 years of death. eFigure 1 in Supplement 1 shows the inclusion and exclusion criteria for the study cohort. For each patient, we measured the following: (1) age, sex, place of residence,^21^ and material deprivation^16,17^ at the date of death (index date); (2) cancer stage at the date of most recent cancer diagnosis^22^; (3) number of comorbidities, using the Ambulatory Case Mix Groups system from Johns Hopkins Medicine, within 1 year preceding death^23^; and (4) COVID-19 test results in the last 30 days of life. Race and ethnicity were not evaluated as they are not systematically collected public health data in Ontario. Material deprivation status was measured using Ontario Marginalization Index quintiles, comparing areas of greatest (quintile 5 [Q5]), intermediate (Q3), and least (Q1) deprivation (eTable 3 in Supplement 1). The data analysis was performed between March and November 2023.

### Exposure and Outcomes

The study exposure was Ontario-wide COVID-19–related hospital entrance screening and visitor restrictions, which started on March 16, 2020.^24,25^ Using this date, we divided the study period into pre–COVID-19 (March 16, 2015, to March 15, 2020) and COVID-19 (March 16, 2020, to March 15, 2021) periods. The primary outcome was place of death as defined according to methods used in previous studies from Ontario.^26,27^ We first identified deaths in hospital inpatient settings (from the Discharge Abstract Database), followed by deaths in the emergency department (from the National Ambulatory Care Reporting System) and long-term-care facilities (from the Continuing Care Reporting System); all remaining deaths were classified as home deaths (eTable 4 in Supplement 1). Emergency department deaths (5979 [3.4%]) and long-term-care facility deaths (3442 [2%]) constituted approximately 5% of all deaths; therefore, we combined the former with hospital inpatient deaths and the latter with home deaths. We then defined the outcome of home death as a binary variable (yes or no) and calculated it as the percentage of patients in the study sample who died at home.

We measured delivery of SPC at the end of life using physician billing claims codes in the Ontario Health Insurance Plan database, consistent with other studies (eTable 4 in Supplement 1).^27,28^ For each patient, we looked back 30 days from the date of death (included in the lookback window) and identified billing claims codes representing an in-person or virtual SPC visit. We defined SPC at the end of life as the percentage of patients who had 1 or more SPC visits in the last 30 days of life.

### Statistical Analysis

#### Descriptive Statistics

Summary statistics were used to describe patient characteristics according to material deprivation quintile in the pre–COVID-19 and COVID-19 periods. We used the standardized difference to examine potential imbalances in patient characteristics according to the stratification variables (material deprivation and study period).^29,30^ Standardized difference was defined as the difference in means or proportions divided by the SD; a standardized difference of 0.1 or higher signified an imbalance between the 2 groups on a specific characteristic.

#### Interrupted Time Series Analysis

We conducted an interrupted time series (ITS) analysis to test the immediate and delayed association of the COVID-19 pandemic with monthly trends of home deaths before and after the onset of the pandemic.^31,32^ Within the ITS framework, we used segmented linear regression, dividing the study period into pre–COVID-19 and COVID-19 segments, and estimated separate intercepts and slopes for each segment.^31,32,33^ The choice of linear regression was contingent on the distribution of the outcome variable and the hypothesized association of interruption with the outcome.^34^ We estimated the immediate association with home deaths in the first month of the pandemic (level change), average percent change in home deaths per month before the pandemic (pre–COVID-19 trend), and change in monthly home deaths after the pandemic compared with the segment preceding the pandemic (slope change) (eTable 5 in Supplement 1). We hypothesized that in the absence of the pandemic, the trend of home deaths in the pre–COVID-19 period would have continued unchanged after the exposure start date (March 16, 2020). We assessed the validity of segmented regression models by examining the plots of studentized residuals, autocorrelation, and partial autocorrelation function and adjusted each model for autocorrelation and seasonal variation (or seasonality) in the study outcome.^32^ The Durbin-Watson statistic was used to assess the magnitude of autocorrelation, where a value of less than 2, 2, and greater than 2 indicated positive, no, and negative autocorrelation, respectively.

We stratified the ITS analysis by material deprivation quintile. Sensitivity analyses were performed by excluding deaths in long-term-care facilities from home deaths (sensitivity analysis 1), excluding patients who tested positive for COVID-19 in the last 30 days of life (sensitivity analysis 2), and excluding patients who had stage I or II cancers (sensitivity analysis 3). Furthermore, subgroup analyses were performed according to the delivery of SPC in the last 30 days of life.

For segmented regression analysis, a 2-sided *P* < .05 was considered statistically significant. Analyses were performed using R, version 4.2.3 (R Foundation for Statistical Computing) and SAS, version 9.4 (SAS Institute Inc).

## Results

### Descriptive Statistics

The study cohort included 173 915 adults who died with cancer (mean [SD] age, 72.1 [12.5] years), of whom 83.7% (95% CI, 83.6%-83.9%) died in the pre–COVID-19 period and 16.3% (95% CI, 16.1%-16.4%) in the COVID-19 period. Males comprised 54.1% (95% CI, 53.8%-54.3%) of the cohort and females, 45.9% (95% CI, 45.7%-46.2%). Overall, 86.0% (95% CI, 85.8%-86.2%) of the cohort resided in an urban area, and 49.6% (95% CI, 49.3%-50.0%) had stage IV cancer. Table 1 presents patient characteristics for the entire cohort and according to material deprivation quintile within the pre–COVID-19 and COVID-19 periods. Overall, 54.5% (95% CI, 54.2%-54.7%) of patients died at home (including 2.0% long-term-care deaths) during the 6-year study period, and 57.8% (95% CI, 57.5%-58.0%) received SPC at the end of life. In the pre–COVID-19 period, patients in Q5 were less likely than those in Q3 and Q1 to die at home (50.6% [95% CI, 50.1%-51.2%] vs 53.9% [95% CI, 53.3%-54.5%] vs 57.0% [95% CI, 56.4%-57.6%], respectively) and to receive SPC at the end of life (55.0% [95% CI, 54.4%-55.5%] vs 57.9% [95% CI, 57.3%-58.5%] vs 63.6% [95% CI, 63.0%-64.2%], respectively). These differences persisted during the COVID-19 period, as shown by the standardized difference estimates (pre–COVID-19 period, 0.058, 0.176, and 0.117; COVID-19 period, 0.041, 0.173, and 0.131, respectively). In both the pre–COVID-19 and COVID-19 periods, patients in Q5 were also more likely to be younger than those in Q3 and Q1, whereas the likelihood of urban residence in Q5 was greater (88.9% and 88.7% for pre–COVID-19 and COVID-19 periods, respectively) than for Q3 (83.0% and 83.6%, respectively) but less than for Q1 (92.6% and 92.8%, respectively).

**Table 1.** Patient Characteristics Within Each Study Period by Material Deprivation Quintile

Characteristic	Pre–COVID-19 period^a^							COVID-19 period^a^							
	No. (%)				Standardized difference			No. (%)					Standardized difference		
	All patients (n = 145 653)^b^	Q1 (n = 26 797)	Q3 (n = 27 958)	Q5 (n = 31 717)	Q1 vs Q3	Q1 vs Q5	Q3 vs Q5	All patients (n = 28 262)^b^	Q1 (n = 5346)	Q3 (n = 5459)	Q5 (n = 6100)	Q1 vs Q3		Q1 vs Q5	Q3 vs Q5
Age group, y															
18-39	2008 (1.4)	415 (1.5)	349 (1.2)	470 (1.5)	0.026	0.005	0.020	398 (1.4)	82 (1.5)	81 (1.5)	87 (1.4)	0.004		0.009	0.005
40-49	4516 (3.1)	877 (3.3)	891 (3.2)	1009 (3.2)	0.005	0.005	0.001	782 (2.8)	167 (3.1)	138 (2.5)	173 (2.8)	0.036		0.017	0.019
50-59	15 888 (10.9)	2623 (9.8)	2922 (10.5)	4015 (12.7)	0.022	0.091	0.069	2997 (10.6)	514 (9.6)	529 (9.7)	744 (12.2)	0.003		0.083	0.080
60-69	32 962 (22.6)	5642 (21.1)	6330 (22.6)	7746 (24.4)	0.038	0.080	0.042	6583 (23.3)	1149 (21.5)	1233 (22.6)	1597 (26.2)	0.026		0.110	0.084
70-79	42 124 (28.9)	7764 (29.0)	8126 (29.1)	9018 (28.4)	0.002	0.012	0.014	8816 (31.2)	1686 (31.5)	1731 (31.7)	1883 (30.9)	0.004		0.014	0.018
≥80	48 155 (33.1)	9476 (35.4)	9340 (33.4)	9459 (29.8)	0.041	0.118	0.077	8686 (30.7)	1748 (32.7)	1747 (32.0)	1616 (26.5)	0.015		0.136	0.121
Sex															
Female	66 985 (46.0)	12 155 (45.4)	12 724 (45.5)	14 918 (47.0)	0.003	0.034	0.031	12 900 (45.6)	2416 (45.2)	2421 (44.3)	2836 (46.5)	0.017		0.026	0.043
Male	78 668 (54.0)	14 642 (54.6)	15 234 (54.5)	16 799 (53.0)	0.003	0.031	0.030	15 362 (54.4)	2930 (54.8)	3038 (55.7)	3264 (53.5)	0.017		0.026	0.043
Residence															
Urban^c^	125 237 (86.2)	24 823 (92.6)	23 196 (83.0)	28 187 (88.9)	0.299	0.130	0.170	24 345 (86.4)	4959 (92.8)	4565 (83.6)	5413 (88.7)	0.286		0.139	0.149
Unknown	384	NA	NA	NA	NA	NA	NA	89	NA	NA	NA	NA		NA	NA
Cancer stage^c^															
I	9446 (12.2)	1657 (11.8)	1837 (12.5)	2121 (12.1)	0.023	0.012	0.011	1769 (14.6)	334 (14.2)	316 (14.0)	420 (15.3)	0.008		0.030	0.038
II	12 306 (15.9)	2323 (16.5)	2319 (15.8)	2633 (15.1)	0.019	0.039	0.019	1890 (15.6)	392 (16.7)	346 (15.3)	402 (14.7)	0.039		0.056	0.018
III	17 042 (22.0)	3056 (21.7)	3274 (22.3)	3814 (21.8)	0.014	0.004	0.011	2721 (22.4)	517 (22.1)	512 (22.7)	628 (22.9)	0.014		0.021	0.006
IV	38 748 (50.0)	7064 (50.1)	7275 (49.5)	8906 (51.0)	0.013	0.017	0.030	5764 (47.5)	1101 (47.0)	1086 (48.0)	1290 (47.1)	0.022		0.002	0.019
Unknown	68 111	12 697	13 253	14 243	NA	NA	NA	16 118	3002	3199	3360	NA		NA	NA
Comorbidity burden in last year of life															
Low (0-5)	12 561 (8.6)	2311 (8.6)	2540 (9.1)	2564 (8.1)	0.016	0.020	0.036	2947 (10.4)	580 (10.9)	567 (10.4)	616 (10.1)	0.015		0.025	0.010
Medium (6-9)	46 762 (32.1)	8464 (31.6)	9018 (32.3)	10 139 (32.0)	0.014	0.008	0.006	8816 (31.2)	1647 (30.8)	1721 (31.5)	1904 (31.2)	0.015		0.009	0.007
High (≥10)	86 330 (59.3)	16 022 (59.8)	16 400 (58.7)	19 014 (59.9)	0.023	0.003	0.026	16 499 (58.4)	3119 (58.3)	3171 (58.1)	3580 (58.7)	0.005		0.007	0.012
COVID-19 status at death^c^^,^^d^															
Negative	NA	NA	NA	NA	NA	NA	NA	13 893 (94.3)	2577 (94.3)	2664 (94.5)	3068 (93.8)	0.006		0.024	0.030
Positive	NA	NA	NA	NA	NA	NA	NA	678 (4.6)	128 (4.7)	123 (4.4)	167 (5.1)	0.016		0.019	0.035
Other^e^	NA	NA	NA	NA	NA	NA	NA	161 (1.1)	27 (1.0)	33 (1.2)	37 (1.1)	0.018		0.014	0.004
Unknown	NA	NA	NA	NA	NA	NA	NA	13 530	2614	2639	2828	NA		NA	NA
Death at home	77 858 (53.5)	15 270 (57.0)	15 069 (53.9)	16 055 (50.6)	0.062	0.128	0.066	16 888 (59.8)	3392 (63.4)	3290 (60.3)	3455 (56.6)	0.066		0.139	0.074
SPC at end of life	84 017 (57.7)	17 037 (63.6)	16 180 (57.9)	17 436 (55.0)	0.117	0.176	0.058	16 445 (58.2)	3426 (64.1)	3149 (57.7)	3394 (55.6)	0.131		0.173	0.041

Abbreviations: NA, not applicable; Q1, least deprived quintile; Q3, intermediate deprivation quintile; Q5, most deprived quintile; SPC, specialized palliative care.

^a^ Pre–COVID-19 period, March 16, 2015, to March 15, 2020; COVID-19 period, March 16, 2020, to March 15, 2021.

^b^ Includes material deprivation quintiles Q1 to Q5.

^c^ Percentages do not account for unknown data.

^d^ COVID-19 test results in last 30 days of life.

^e^ Includes indeterminate, canceled, and rejected test results.

Table 2 shows a comparison of patient characteristics within material deprivation quintiles Q1, Q3, and Q5 by study period. For the full study cohort, the proportion of patients dying at home increased from 53.5% (95% CI, 53.2%-53.7%) in the pre–COVID-19 period to 59.8% (59.2%-60.3%) in the COVID-19 period, while the percentage of patients who received SPC at the end of life did not change (57.7% [95% CI, 57.4%-57.9%] and 58.2% [95% CI, 57.6%-58.8%], respectively). Comparison of the 2 study periods revealed that within each material deprivation quintile, home deaths increased in the COVID-19 period in all 3 quintiles, reaching 56.6% (95% CI, 55.4%-57.9%) in Q5, 60.3% (95% CI, 59.0%-61.6%) in Q3, and 63.4% (95% CI, 62.2%-64.7%) in Q1.

**Table 2.** Patient Characteristics Within Material Deprivation Quintiles by Study Period

Characteristic	All patients (n = 173 915)^a^			Q1 (n = 32 143)			Q3 (n = 33 417)			Q5 (n = 37 817)		
	No. (%)		Standardized difference	No. (%)		Standardized difference	No. (%)		Standardized difference	No. (%)		Standardized difference
	Before COVID-19 (n = 145 653)^b^	During COVID-19 (n = 28 262)^b^		Before COVID-19 (n = 26 797)^b^	During COVID-19 (n = 5346)^b^		Before COVID-19 (n = 27 958)^b^	During COVID-19 (n = 5459)^b^		Before COVID-19 (n = 31 717)^b^	During COVID-19 (n = 6100)^b^	
Age group, y												
18-39	2008 (1.4)	398 (1.4)	0.003	415 (1.5)	82 (1.5)	0.001	349 (1.2)	81 (1.5)	0.020	470 (1.5)	87 (1.4)	0.005
40-49	4516 (3.1)	782 (2.8)	0.020	877 (3.3)	167 (3.1)	0.008	891 (3.2)	138 (2.5)	0.040	1009 (3.2)	173 (2.8)	0.020
50-59	15 888 (10.9)	2997 (10.6)	0.010	2623 (9.8)	514 (9.6)	0.006	2922 (10.5)	529 (9.7)	0.025	4015 (12.7)	744 (12.2)	0.014
60-69	32 962 (22.6)	6583 (23.3)	0.016	5642 (21.1)	1149 (21.5)	0.011	6330 (22.6)	1233 (22.6)	0.001	7746 (24.4)	1597 (26.2)	0.040
70-79	42 124 (28.9)	8816 (31.2)	0.050	7764 (29.0)	1686 (31.5)	0.056	8126 (29.1)	1731 (31.7)	0.058	9018 (28.4)	1883 (30.9)	0.053
≥80	48 155 (33.1)	8686 (30.7)	0.050	9476 (35.4)	1748 (32.7)	0.056	9340 (33.4)	1747 (32.0)	0.030	9459 (29.8)	1616 (26.5)	0.074
Sex												
Female	66 985 (46.0)	12 900 (45.6)	0.007	12 155 (45.4)	2416 (45.2)	0.003	12 724 (45.5)	2421 (44.3)	0.023	14 918 (47.0)	2836 (46.5)	0.011
Male	78 668 (54.0)	15 362 (54.4)	0.007	14 642 (54.6)	2930 (54.8)	0.003	15 234 (54.5)	3038 (55.7)	0.023	16 799 (53.0)	3264 (53.5)	0.011
Residence												
Urban	125 237 (86.2)	24 345 (86.4)	0.005	24 823 (92.6)	4959 (92.8)	0.005	23 196 (83.0)	4565 (83.6)	0.018	28 187 (88.9)	5413 (88.7)	0.004
Unknown	384	89	NA	NA	NA	NA	NA	NA	NA	NA	NA	NA
Cancer stage												
I	9446 (12.2)	1769 (14.6)	0.070	1657 (11.8)	334 (14.2)	0.074	1837 (12.5)	316 (14.0)	0.044	2121 (12.1)	420 (15.3)	0.093
II	12 306 (15.9)	1890 (15.6)	0.008	2323 (16.5)	392 (16.7)	0.007	2319 (15.8)	346 (15.3)	0.013	2633 (15.1)	402 (14.7)	0.011
III	17 042 (22.0)	2721 (22.4)	0.010	3056 (21.7)	517 (22.1)	0.009	3274 (22.3)	512 (22.7)	0.009	3814 (21.8)	628 (22.9)	0.026
IV	38 748 (50.0)	5764 (47.5)	0.050	7064 (50.1)	1101 (47.0)	0.063	7275 (49.5)	1086 (48.0)	0.028	8906 (51.0)	1290 (47.1)	0.078
Unknown	68 111	16 118		12 697	3002		13 253	3199		14 243	3360	
Comorbidity burden in last year of life												
Low (0-5)	12 561 (8.6)	2947 (10.4)	0.061	2311 (8.6)	580 (10.9)	0.075	2540 (9.1)	567 (10.4)	0.044	2564 (8.1)	616 (10.1)	0.070
Medium (6-9)	46 762 (32.1)	8816 (31.2)	0.020	8464 (31.6)	1647 (30.8)	0.017	9018 (32.3)	1721 (31.5)	0.016	10 139 (32.0)	1904 (31.2)	0.016
High (≥10)	86 330 (59.3)	16 499 (58.4)	0.018	16 022 (59.8)	3119 (58.3)	0.029	16 400 (58.7)	3171 (58.1)	0.012	19 014 (59.9)	3580 (58.7)	0.026
Death at home	77 858 (53.5)	16 888 (59.8)	0.127	15 270 (57.0)	3392 (63.4)	0.132	15 069 (53.9)	3290 (60.3)	0.129	16 055 (50.6)	3455 (56.6)	0.121
SPC at end of life	84 017 (57.7)	16 445 (58.2)	0.010	17 037 (63.6)	3426 (64.1)	0.011	16 180 (57.9)	3149 (57.7)	0.004	17 436 (55.0)	3394 (55.6)	0.013

Abbreviations: NA, not applicable; Q1, least deprived quintile; Q3, intermediate deprivation quintile; Q5, most deprived quintile; SPC, specialized palliative care.

^a^ Includes material deprivation quintiles Q1 to Q5.

^b^ Percentages do not account for unknown data.

### Trends of Home Deaths and SPC Delivery in the Pre–COVID-19 and COVID-19 Periods

**Figure 1.**
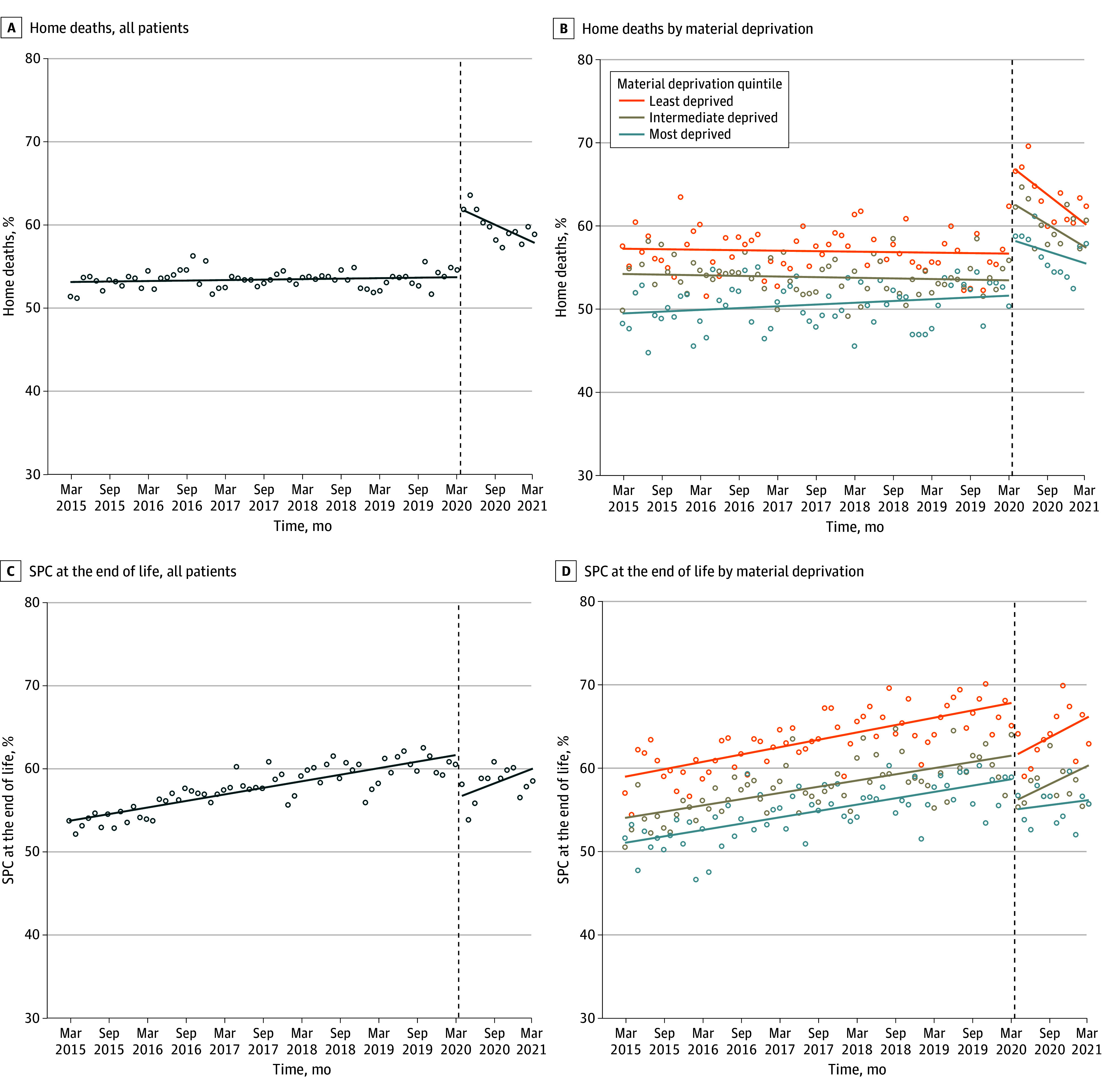
Monthly Rates of Death at Home and Delivery of Specialized Palliative Care (SPC) at the End of Life for All Patients and by Material Deprivation Quintile Dashed lines indicate the start of the COVID-19 pandemic on March 16, 2020; dots, observed rates; solid lines, fitted estimates from interrupted time series analysis without seasonal variation to reveal the underlying trend.

eFigure 2 in Supplement 1 shows the absolute number of deaths per month for the entire study period. During the 5-year pre–COVID-19 period, monthly rates of home deaths were stable in the full cohort and for Q1 and Q3. Home deaths increased slightly for Q5 during this period but remained lower than for Q3 or Q1 (Table 3; Figure 1A and B). For the full cohort, there was an immediate increase in home deaths in the first month of the COVID-19 pandemic (level change, 8.3%; 95% CI, 7.4%-9.1%); however, this increase was less marked in Q5 (6.1%; 95% CI, 4.4%-7.8%) compared with Q1 (11.4%; 95% CI, 9.6%-13.2%) and Q3 (10.0%; 95% CI, 9.0%-11.1%). After the initial rapid increase, the monthly rates of home deaths gradually decreased for all 3 quintiles in the first year of the pandemic; however, the rates continued to be higher compared with the pre–COVID-19 period and remained highest in Q1 and lowest in Q5.

**Table 3.** Changes in Monthly Trends of Study Outcomes at the Start of, Before, and During the COVID-19 Pandemic

Parameter^a^	Monthly trend, % per mo (95% CI)			
	All patients^b^	Q1	Q3	Q5
Home death				
Level change at start of COVID-19 pandemic	8.3 (7.4 to 9.1)^c^	11.4 (9.6 to 13.2)^c^	10.0 (9.0 to 11.1)^c^	6.1 (4.4 to 7.8)^d^
Trend before COVID-19 pandemic	0.013 (0.001 to 0.025)	−0.010 (−0.026 to 0.006)	−0.018 (−0.028 to −0.009)	0.040 (0.021 to 0.059)^e^
Slope change during COVID-19 pandemic	−0.355 (−0.458 to −0.252)^d^	−0.683 (−0.899 to −0.468)^d^	−0.503 (−0.638 to −0.367)^d^	−0.210 (−0.406 to −0.014)
Delivery of SPC at end of life				
Level change at start of COVID-19 pandemic	−5.3 (−6.3 to −4.4)^c^	−6.6 (−8.1 to −5.1)^c^	−5.6 (−7.4 to −4.1)^d^	−4.1 (−5.3 to −3.0)^d^
Trend before COVID-19 pandemic	0.133 (0.122 to 0.144)^c^	0.149 (0.131 to 0.167)^c^	0.126 (0.107 to 0.144)^c^	0.130 (0.118 to 0.143)^c^
Slope change during COVID-19 pandemic	0.191 (0.076 to 0.305)	0.275 (0.097 to 0.452)	0.254 (0.061 to 0.447)	0.006 (−1.128 to 0.141)
Home death, SPC at end of life				
Level change at start of COVID-19 pandemic	6.6 (5.4 to 7.8)^c^	7.6 (5.4 to 9.7)^c^	9.0 (7.2 to 10.7)^c^	1.2 (−1.0 to 3.5)
Trend before COVID-19 pandemic	0.017 (−0.001 to 0.035)	−0.011 (−0.034 to 0.012)	−0.018 (−0.034 to −0.002)	0.055 (0.025 to 0.084)
Slope change during COVID-19 pandemic	−0.312 (−0.459 to −0.165)^e^	−0.378 (−0.631 to −0.125)	−0.477 (−0.683 to −0.270)^e^	0.244 (−0.020 to 0.507)
Home death, no SPC at end of life				
Level change at start of COVID-19 pandemic	12.6 (11.6 to 13.7)^c^	17.5 (15.2 to 19.8)^c^	12.7 (10.8 to 14.5)^c^	13.9 (11.9 to 15.8)^c^
Trend before COVID-19 pandemic	−0.043 (−0.054 to −0.031)^d^	−0.075 (−0.112 to −0.038)	−0.070 (−0.087 to −0.052)^d^	−0.026 (−0.042 to −0.009)
Slope change during COVID-19 pandemic	−0.503 (−0.621 to −0.385)^c^	−1.003 (−1.264 to −0.743)^d^	−0.501 (−0.729 to −0.274)^e^	−0.786 (−1.023 to −0.549)^d^

Abbreviations: Q1, least deprived quintile; Q3, intermediate deprivation quintile; Q5, most deprived quintile; SPC, specialized palliative care.

^a^ Level change represents an immediate effect of the COVID-19 pandemic (the interruption on March 16, 2020) on home death (outcome) in the first month of the pandemic. Trend before COVID-19 pandemic represents an average percent change in home deaths per month before the start of the COVID-19 pandemic. Slope change during COVID-19 pandemic represents an average percent change in home deaths per month during the COVID-19 pandemic compared with the segment preceding the pandemic.

^b^ Includes material deprivation quintiles Q1 to Q5.

^c^ *P* < .001.

^d^ *P* < .01.

^e^ *P* < .05.

Monthly rates of SPC delivery during the 5-year pre–COVID-19 period were lowest in Q5 and highest in Q1, with a steady increase over that period that was similar for all 3 material deprivation quintiles (Table 3; Figure 1C and D). At the onset of the pandemic, there was an immediate decrease in level of delivery of SPC of 5.3% (95% CI, −6.3% to −4.4%), with no significant difference in this change among material deprivation quintiles. After this initial rapid decrease, there was an increase in the monthly rates of SPC delivery, which was higher for patients in Q1 and Q3 than in Q5.

**Figure 2.**
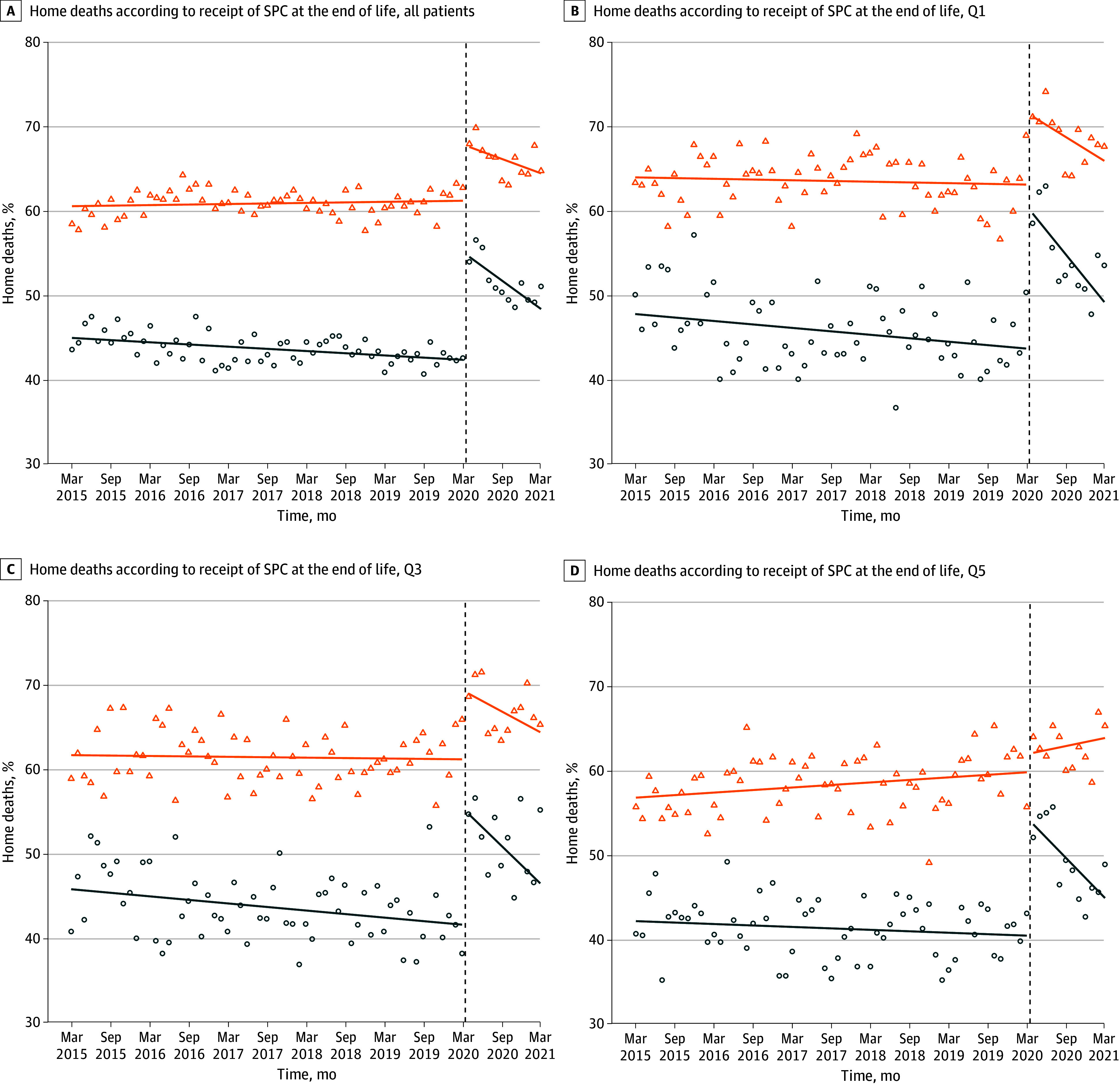
Monthly Rates of Death at Home by Receipt of Specialized Palliative Care (SPC) at the End of Life for All Patients and by Material Deprivation Quintile The solid lines indicate fitted estimates from interrupted time series analysis without seasonal variation to reveal the underlying trend. The orange lines represent SPC; blue lines represent no SPC. The triangles and dots indicate observed rates for SPC and no SPC, respectively. Dashed lines indicate the start of the COVID-19 pandemic on March 16, 2020. Q1 indicates the least deprived quintile; Q3, intermediate deprivation quintile; Q5, most deprived quintile.

The subgroup analysis of home deaths according to delivery of SPC at the end of life is shown in Table 3 and Figure 2. For the full study cohort, patients who received SPC were more likely to die at home than those who did not receive SPC both in the pre–COVID-19 period (60.8% [95% CI, 60.4%-61.1%] vs 43.5% [95% CI, 43.1%-43.9%]) and in the COVID-19 period (65.8% [95% CI, 65.1%-66.5%] vs 51.3% [95% CI, 50.4%-52.3%]). However, the increase in home deaths at the onset of the pandemic was greater for those who did not receive SPC than for those who received SPC. For patients in Q1, the level change was 17.5% (95% CI, 15.2%-19.8%) for those who received no SPC vs 7.6% (95% CI, 5.4%-9.7%) for those who received SPC (Figure 2B). For Q3, the corresponding level changes were 12.7% (95% CI, 10.8%-14.5%) and 9.0% (95% CI, 7.2%-10.7%), respectively (Figure 2C). For patients in Q5, the level change was significant for patients who received no SPC at the end of life (13.9%; 95% CI, 11.9%-15.8%) and not significant for those who received SPC at the end of life (1.2%; 95% CI, −1.0% to 3.5%) (Figure 2D).

The 3 sensitivity analyses excluding the long-term-care deaths from home deaths, patients who tested positive for COVID-19 at the end of life, and stage I and II cancers are shown in eTables 6 to 8 in Supplement 1, respectively. All yielded similar results.

## Discussion

In this population-based cohort study of more than 170 000 patients who died with cancer, we evaluated the association of the COVID-19 pandemic with trends of home death and delivery of SPC and investigated potential disparities according to SES. During the 5 years before the pandemic, deaths at home remained constant, while rates of SPC increased. At the onset of the pandemic, deaths at home increased rapidly, with a concomitant decrease in delivery of SPC at the end of life. Patients with the lowest SES were consistently the least likely to die at home and to receive SPC at the end of life. Moreover, those with the lowest SES had a comparatively smaller increase in home deaths at the onset of the pandemic, which was observed only among patients who did not receive SPC at the end of life. These findings suggest that the COVID-19 pandemic was associated with a worsened end-of-life experience and amplified socioeconomic disparities for patients with advanced cancer.

The surge in deaths at home at the onset of the pandemic may have been largely influenced by patients’ efforts to avoid death in an inpatient setting. In a survey of more than 700 bereaved people in the UK, deaths in the hospital during the COVID-19 pandemic were associated with an increased likelihood of poorer end-of-life experiences compared with deaths at home.^35^ Several qualitative studies have described the distress of patients and caregivers who experienced end-of-life care in the inpatient setting during the COVID-19 pandemic.^36,37,38^ Reasons for this distress included social isolation and loneliness related to strict visitor policies; inconsistency in these policies; lack of family involvement in end-of-life decision making; feeling unsafe in the hospital due to the risk of COVID-19 infection; and lack of support from an understaffed group of health care professionals, while family members were not allowed to help with feeding and other personal care. While many patients chose to die at home during the pandemic, this choice may have been less viable for those with lower SES due to a lack of resources to pay for private personal support; less access to suitable, safe, and stable housing; less access to SPC services; and lack of a support network of informal caregivers with the capacity to take time off from work and advocate for the patient’s needs.^39,40^

The surge in deaths at home was accompanied by a simultaneous decrease in SPC delivery at the end of life. This abrupt drop in SPC delivery at the onset of the COVID-19 pandemic followed a decade of a gradual increase of SPC delivery in Ontario^26^ and may have been associated with delayed or lacking SPC access resulting from COVID-19 restrictions, staffing shortages due to nurses and physicians contracting COVID-19, or redeployment of palliative care staff to help with the care of patients with COVID-19.^41,42^ Both leading up to the pandemic and during its first year, we found that patients with lower SES were less likely to receive SPC at the end of life than those with higher SES. The persistent nature of this disparity in SPC delivery is shown by its existence in studies describing care in Ontario between 2004 and 2015^15,26,43^ as well as in studies taking place in other high-income countries.^13^ The mechanisms for inequality are not well characterized but may include local availability of services, awareness of these services, and ability to advocate for them.

The simultaneous increase in home deaths and decrease in SPC delivery meant that surging home deaths during the COVID-19 pandemic were often not supported by SPC. Indeed, for patients with the lowest SES, home deaths increased only among those without SPC support during the month before death. A similar inequity was demonstrated in a US study: for Medicaid-insured (but not commercially insured) patients with advanced cancer, place of death shifted from hospital to home without hospice during the first 4 months of the COVID-19 pandemic.^44^ The relative increase in home deaths unsupported by SPC or hospice among socioeconomically disadvantaged populations during the COVID-19 pandemic could be due to patients wanting to avoid inpatient care but lacking access to or knowledge of end-of-life resources that would normally facilitate and support a home death. Similar to critical care, a plan for responding to future crises should be put into place for palliative care. This plan should include care for patients with cancer and other serious illnesses and should prioritize marginalized patients to avoid systemic inequity in times of health system strain.^45,46^

### Strengths and Limitations

A strength of our study is the ITS design, which allowed us to quantify the magnitude of the immediate and delayed association of the COVID-19 pandemic with home deaths and SPC delivery and, through stratification, to test the association of material deprivation status and SPC with this change. We used robust population-based data linked at the individual level and including full population coverage of all adult deaths across all places of death in Ontario.

Our study also has limitations. First, we did not directly assess the quality of death at home or the preferred place of death. Death at home may not be desired or even optimal, and the preferred site of death may be influenced by many factors, including type and stage of disease, complexity of symptoms, the home environment, family support, and availability of care at home.^5,47^ However, we measured delivery of SPC in the last month of life, which is considered to be a quality indicator for end-of-life cancer care^48,49^ and is in line with current clinical practice guidelines recommending provision of dedicated SPC throughout the disease course.^50,51^

Second, we were unable to identify the number of home deaths in the residential hospice setting because this information is not available in Ontario’s health administrative databases. However, the number is likely small given the dearth of hospice beds available for patients with and without cancer in Ontario (approximately 3.5 hospice beds per 100 000 people).^52^ Third, the Ontario Marginalization Index underestimates material deprivation in certain populations, such as institutionalized individuals who are not counted or Indigenous people living on reserves who may be undercounted on the long-form census.^17^ Fourth, our study reports only on the first year of the COVID-19 pandemic; however, this study interval is, to our knowledge, the longest to date to assess disparities in end-of-life care during the pandemic. Fifth, we were not able to examine outcomes by racial and ethnic identity because this information is not systematically collected in the public health data of Ontario. Further cohort studies should examine intersectional disparities in end-of-life outcomes among patients with cancer in Canada and globally. Ongoing qualitative research with bereaved family members of patients who died of cancer during the pandemic will provide subjective accounts of care received and preferences for care.

## Conclusions

In this cohort study with an ITS analysis, the COVID-19 pandemic was associated with a surge in deaths at home and a concomitant decrease in SPC at the end of life. Among patients with the lowest SES, the increase in home deaths was comparatively smaller and occurred only among those who did not receive SPC at the end of life. Future research should focus on understanding mechanisms for these disparities and developing clinical and policy interventions to ensure consistent and equitable access to SPC, particularly during times of crisis.

## Notes

Supplement 1. **eTable 1.** Description of Health Administrative Databases Used in the Study **eTable 2.** STROBE Statement: Checklist of Items That Should Be Included in Reports of Cohort Studies **eTable 3.** Defining Material Deprivation **eTable 4.** Defining Place of Death and Specialized Palliative Care **eTable 5.** The Interrupted Time-Series Analysis **eTable 6.** Changes in Monthly Trends of Study Outcomes at Start of, Before, and During the COVID-19 Pandemic, Sensitivity Analysis 1, Excluding Long-Term-Care Facility Deaths From Home Deaths **eTable 7.** Changes in Monthly Trends of Study Outcomes at Start of, Before, and During the COVID-19 Pandemic, Sensitivity Analysis 2, Excluding Patients Who Tested Positive for COVID-19 in the Last 30 Days of Life **eTable 8.** Changes in Monthly Trends of Study Outcomes at Start of, Before, and During the COVID-19 Pandemic, Sensitivity Analysis 3, Excluding Patients Who Died With Stage I and II Cancers **eFigure 1.** Study Cohort **eFigure 2.** Absolute Number of Monthly Deaths, March 16, 2015, to March 15, 2021

Supplement 2.
Data Sharing Statement
