# Supplementary material for: Socioeconomic Status, Palliative Care, and Death at Home Among Patients With Cancer Before and During COVID-19
Source: JAMA Netw Open. 2024 Feb 27;7(2):e240503. doi: 10.1001/jamanetworkopen.2024.0503 (PMC10900963; doi:10.1001/jamanetworkopen.2024.0503)
Supplement: Supplement 1. — eTable 1. Description of Health Administrative Databases Used in the Study eTable 2. STROBE Statement: Checklist of Items That Should Be Included in Reports of Cohort Studies eTable 3. Defining Material Deprivation eTable 4. Defining Place of Death and Specialized Palliative Care eTable 5. The Interrupted Time-Series Analysis eTable 6. Changes in Monthly Trends of Study Outcomes at Start of, Before, and During the COVID-19 Pandemic, Sensitivity Analysis 1, Excluding Long-Term-Care Facility Deaths From Home Deaths eTable 7. Changes in Monthly Trends of Study Outcomes at Start of, Before, and During the COVID-19 Pandemic, Sensitivity Analysis 2, Excluding Patients Who Tested Positive for COVID-19 in the Last 30 Days of Life eTable 8. Changes in Monthly Trends of Study Outcomes at Start of, Before, and During the COVID-19 Pandemic, Sensitivity Analysis 3, Excluding Patients Who Died With Stage I and II Cancers eFigure 1. Study Cohort eFigure 2. Absolute Number of Monthly Deaths, March 16, 2015, to March 15, 2021 [file jamanetwopen-e240503-s001.pdf]

Supplemental Online Content

Iqbal J, Moineddin R, Fowler RA, et al. Socioeconomic status, palliative care, and cancer deaths at home before and during COVID-19. *JAMA Netw Open*. 2024;7(2):e240503. doi:10.1001/jamanetworkopen.2024.0503

eTable 1. Description of Health Administrative Databases Used in the Study

eTable 2. STROBE Statement: Checklist of Items That Should Be Included in Reports of Cohort Studies

eTable 3. Defining Material Deprivation

eTable 4. Defining Place of Death and Specialized Palliative Care

eTable 5. The Interrupted Time Series Analysis

eTable 6. Changes in Monthly Trends of Study Outcomes at Start of, Before, and During the COVID-19 Pandemic, Sensitivity Analysis 1, Excluding Long-Term-Care Facility Deaths From Home Deaths

eTable 7. Changes in Monthly Trends of Study Outcomes at Start of, Before, and During the COVID-19 Pandemic, Sensitivity Analysis 2, Excluding Patients Who Tested Positive for COVID-19 in the Last 30 Days of Life

eTable 8. Changes in Monthly Trends of Study Outcomes at Start of, Before, and During the COVID-19 Pandemic, Sensitivity Analysis 3, Excluding Patients Who Died With Stage I and II Cancers

eFigure 1. Study Cohort

eFigure 2. Absolute Number of Monthly Deaths, March 16, 2015, to March 15, 2021

This supplemental material has been provided by the authors to give readers additional information about their work.

© 2024 Iqbal J et al. *JAMA Network Open.*

eTable 1. Description of Health Administrative Databases Used in the Study

Ontario's health administrative databases are housed at the ICES (formerly Institute for Clinical Evaluative Sciences), Toronto. Ontario's Personal Health Information Protection Act (PHIPA) designates ICES as a prescribed entity, allowing ICES to collect and use health information without individual consent or research ethics approval for analysis and compiling statistical information about the Canadian healthcare system. More details at: https://www.ices.on.ca/data-privacy/

Database	Description	Clinical information
Ontario Cancer Registry (OCR)	OCR contains demographic and clinical information on incident cancers (except non-melanoma skin cancer) and mortality in Ontario since 1964.	Stage of cancer diagnosis
Discharged Abstract Database (DAD)	The DAD captures administrative, clinical and demographic information on hospital discharges (including deaths, sign-outs and transfers).	Place of death
National Ambulatory Care Reporting System metadata (NACRS)	The NACRS contains hospital-based and community-based ambulatory care data, including day surgery, outpatient and community-based clinics, and emergency departments.	Place of death
Continuing Care Reporting System (CCRS)	The CCRS contains demographic, clinical, functional and resource utilization information on individuals receiving continuing care services in hospitals or long-term care homes in Canada.	Place of death
Ontario Marginalization Index (ON-Marg)	ON-Marg is a data tool that combines a wide range of demographic indicators to examine different dimensions of marginalization in Ontario.	Area-level material deprivation quintile
Ontario Health Insurance Plan (OHIP)	OHIP database contains physician claims for the clinical services provided to residents of Ontario.	Specialized palliative care
The Johns Hopkins ACG® System	The Johns Hopkins ACG System is a statistically valid, case-mix methodology used to describe or predict a population's past or future healthcare utilization and costs. The ACG System is also widely used by researchers and analysts to compare various patient populations' prior health resource use while considering morbidity or illness burden.	Comorbidity
Ontario Laboratories Information System (OLIS)	OLIS dataset includes data on laboratory tests covered by OHIP.	COVID-19 test result
Postal Code Conversion File (PCCF)	The PCCF facilitates the linkage of census-based geographic attributes (such as a rural indicator and neighbourhood income quintile) via postal codes.	Place of residence
Registered Persons Database (RPDB)	The RPDB contains demographic data (birth and death dates, gender, and postal codes) and dates of eligibility for healthcare services for every Ontarian with an Ontario Health Insurance Plan.	Age group, Sex, Vital status

© 2024 Iqbal J et al. *JAMA Network Open.*

eTable 2. STROBE Statement: Checklist of Items That Should Be Included in Reports of Cohort Studies

	Item No	Recommendation	Page No
**Title and abstract**	1	(*a*) Indicate the study's design with a commonly used term in the title or the abstract	
		(*b*) Provide in the abstract an informative and balanced summary of what was done and what was found	4
**Introduction**			
Background/rationale	2	Explain the scientific background and rationale for the investigation being reported	6
Objectives	3	State specific objectives, including any prespecified hypotheses	6-7
**Methods**			
Study design	4	Present key elements of study design early in the paper	7
Setting	5	Describe the setting, locations, and relevant dates, including periods of recruitment, exposure, follow-up, and data collection	7
Participants	6	(*a*) Give the eligibility criteria, and the sources and methods of selection of participants. Describe methods of follow-up	7-8
Variables	7	Clearly define all outcomes, exposures, predictors, potential confounders, and effect modifiers. Give diagnostic criteria, if applicable	8
Data sources/ measurement	8*	For each variable of interest, give sources of data and details of methods of assessment (measurement). Describe comparability of assessment methods if there is more than one group	7-8
Bias	9	Describe any efforts to address potential sources of bias	
Study size	10	Explain how the study size was arrived at	
Quantitative variables	11	Explain how quantitative variables were handled in the analyses. If applicable, describe which groupings were chosen and why	9
Statistical methods	12	(*a*) Describe all statistical methods, including those used to control for confounding	9-10
		(*b*) Describe any methods used to examine subgroups and interactions	
		(*c*) Explain how missing data were addressed	
		(*d*) If applicable, explain how loss to follow-up was addressed	
		(*e*) Describe any sensitivity analyses	
**Results**			

© 2024 Iqbal J et al. *JAMA Network Open.*

Participants	13*	(a) Report numbers of individuals at each stage of study—eg numbers potentially eligible, examined for eligibility, confirmed eligible, included in the study, completing follow-up, and analysed	10
		(b) Give reasons for non-participation at each stage	
		(c) Consider use of a flow diagram	
Descriptive data	14*	(a) Give characteristics of study participants (eg demographic, clinical, social) and information on exposures and potential confounders	10-11
		(b) Indicate number of participants with missing data for each variable of interest	
		(c) Summarise follow-up time (eg, average and total amount)	
Outcome data	15*	Report numbers of outcome events or summary measures over time	11
Main results	16	(a) Give unadjusted estimates and, if applicable, confounder-adjusted estimates and their precision (eg, 95% confidence interval). Make clear which confounders were adjusted for and why they were included	11-12
		(b) Report category boundaries when continuous variables were categorized	
		(c) If relevant, consider translating estimates of relative risk into absolute risk for a meaningful time period	
Other analyses	17	Report other analyses done—eg analyses of subgroups and interactions, and sensitivity analyses	12-13
**Discussion**			
Key results	18	Summarise key results with reference to study objectives	13
Limitations	19	Discuss limitations of the study, taking into account sources of potential bias or imprecision. Discuss both direction and magnitude of any potential bias	15
Interpretation	20	Give a cautious overall interpretation of results considering objectives, limitations, multiplicity of analyses, results from similar studies, and other relevant evidence	13-15
Generalisability	21	Discuss the generalisability (external validity) of the study results	15
**Other information**			
Funding	22	Give the source of funding and the role of the funders for the present study and, if applicable, for the original study on which the present article is based	20

*Give information separately for exposed and unexposed groups.

Note: An Explanation and Elaboration article discusses each checklist item and gives methodological background and published examples of transparent reporting. The STROBE checklist is best used in conjunction with this article (freely available on the Web sites of PLoS Medicine at

© 2024 Iqbal J et al. *JAMA Network Open.*

http://www.plosmedicine.org/, Annals of Internal Medicine at http://www.annals.org/, and Epidemiology at http://www.epidem.com/). Information on the STROBE Initiative is available at http://www.strobe-statement.org.

eTable 3. Defining Material Deprivation

Material deprivation is a tool in the Ontario Marginalization Index (ON-Marg) that is closely connected to poverty and it refers to inability for individuals and communities to access and attain basic material needs. The ON-Marg links the postal codes of Ontario individuals to the census tract-level data and assigns all residents a marginalization level depending upon their neighborhoods. The postal codes are then scored, ranked, and categorized into the quintiles Q1 (least deprived) to Q5 (most deprived).

More information at https://www.ontariohealthprofiles.ca/onmargON.php#onmPHOdata2011

Indicator	Data Source	Definition
% lone-parent families	Short Form Census	Numerator: # lone-parent families<br>Denominator: Total # of census families
Proportion of income from government transfer payments	T1FF	Numerator: Median dollar amount from government transfer for census families<br>Denominator: Median dollar amount from all income sources for census families
% below Low Income Measure	T1FF	Numerator: Number of people living in census families earning less than the after-tax low income measure<br>Denominator: Total number of people
% houses in fair or poor condition [2]	MPAC	Numerator: # residential households in fair or poor condition<br>Denominator: Total # of residential households

Abbreviations: T1FF, Statistics Canada T1 Family File; MPAC, Municipal Property Assessment Corporation.

Source: 2011 Ontario Marginalization Index: Technical document

Reference: Matheson, FI; Ontario Agency for Health Protection and Promotion (Public Health Ontario). 2011 Ontario marginalization index: technical document. Toronto, ON: St. Michael's Hospital; 2017. Joint publication with Public Health Ontario.

Footnotes:

[1] MPAC classifies the structures on a property using a range from poor to good. Fair Condition is defined by MPAC as "A structure is in fair condition if it has not received normal maintenance and is showing signs of neglect. The neglect would be sufficient to impede the marketability (resale value) of the house." Poor Condition is defined as "A structure is in poor condition if it has received no maintenance, it has been abused and displays a significant amount of abnormal deterioration, which would require a major expense to cure (fix)."

© 2024 Iqbal J et al. *JAMA Network Open.*

eTable 4. Defining Place of Death and Specialized Palliative Care

**Database (Variable)**	**Code**	**Label**	**Method**
DAD (Discharge Disposition [DISCHDISP])	07	Died (before 2018)	Presence of any of the DISCHDISP codes was considered a hospital death.
	72	Died in facility (starting 2018/19)	
	66	Died while on pass/leave	
	67	Suicide out of facility	
	73	Medical assistance in dying (MAID)	
	74	Suicide in facility	
NACRS (Visit Disposition [VISDISP])	10	Death after arrival	Presence of any of the VISDISP codes was considered an emergency department death.
	11	Death on arrival (includes in ER).	
	71	Dead on arrival (starting 2018/19)	
	72	Died in facility (starting 2018/19)	
	73	Medical assistance in dying (MAID)	
	74	Suicide in facility	
	10	Death after arrival	
CCRS (Discharge to Faculty Type [DISCHARGE_TO_FACILITY_TYPE])	11	Deceased	Not applicable
OHIP (Fee Code [feecode])	A945	Special palliative care consultation in clinic, office, home; minimum 50 min	Each billing of A945 is considered a specialized palliative care consultation in the outpatient.
	C945	Special palliative care consultation	Each billing of C945 is considered a specialized palliative care consultation in the inpatient.
	K023	Palliative care support in half hour increments; may be used to add time for longer consultations following a code for A945, or for any PC support visit	At least two billings of K023 codes by the same physician, and the second billing within six months of the first billing is considered specialized palliative care consultation.

© 2024 Iqbal J et al. *JAMA Network Open.*

eTable 5. The Interrupted Time Series Analysis

The formatted time series dataset looks like this:

Y	Time (T)	Intervention (D)	Time since (P)
10	1	0	0
12	2	0	0
14	3	0	0
11	4	0	0
15	6	1	1
18	7	1	2
18	8	1	3
20	9	1	4

Intervention

The basic segmented regression model equation is:

$$Y = \beta_0 + \beta_1 T + \beta_2 (D) + \beta_3 (P) + e$$

Where:

Y is the study outcome at time ‘*t*’;
‘*T*’ is a continuous variable which indicates time (as months) passed from the start of the observation period;
‘*D*’ is a dummy variable taking the values ‘0’ for pre-intervention and ‘1’ for post-intervention segment;
‘*P*’ is a continuous variable taking the values ‘0’ in the pre-intervention and counting the months in the post-intervention segment at time ‘*t*’;
β_0 is the base level of the outcome (the intercept or constant);
β_1 is the slope of the line before the intervention;
β_2 is the immediate effect that occurs after the intervention;
β_3 is the difference between the slope of the line before and the slope of the line after the intervention, and It represents the sustained effect of the intervention.
e is the error term.

© 2024 Iqbal J et al. *JAMA Network Open.*

eTable 6. Changes in Monthly Trends of Study Outcomes at Start of, Before, and During the COVID-19 Pandemic, Sensitivity Analysis 1, Excluding Long-Term Care Facility Deaths from Home Deaths

Outcome	Parameter [a]	Monthly Trend, % per month (95% CI)			
		All Patients [b]	Least Deprived Quintile (Q1)	Intermediate Deprived Quintile (Q3)	Most Deprived Quintile (Q5)
Home death	Level change at start of COVID-19 pandemic	7.7 (7.0, 8.5) ***	10.3 (8.5, 12.0) ***	9.2 (8.0, 10.4) ***	5.7 (3.9, 7.4) **
	Trend before COVID-19 pandemic	0.02 (0.01, 0.03)*	0.01 (-0.01, 0.03)	-0.01 (-0.02, 0.001)	0.1 (0.04, 0.1) **
	Slope change during COVID-19 pandemic	-0.3 (-0.4, -0.2) **	-0.6 (-0.8, -0.4) **	-0.4 (-0.5, -0.3) **	-0.2 (-0.4, 0.04)
Home death, Received SPC at end-of-life	Level change at start of COVID-19 pandemic	7.1 (5.9, 8.3) ***	7.8 (5.7, 9.9) **	9.0 (7.5, 10.6) ***	1.0 (-1.2, 0.1)
	Trend before COVID-19 pandemic	0.02 (-0.001, 0.03)	-0.01 (-0.03, 0.02)	-0.01 (-0.03, 0.001)	0.1 (0.02, 0.1)
	Slope change during COVID-19 pandemic	-0.4 (-0.5, -0.2) *	-0.4 (-0.6, -0.1)	-0.5 (-0.7, -0.3) *	0.3 (0.03, 0.6)
Home death, No SPC at end-of-life	Level change at start of COVID-19 pandemic	11.6 (10.8, 12.4) ***	17.3 (15.3, 19.3) ***	12.7 (10.6, 14.8) ***	14.0 (12.3, 15.6) ***
	Trend before COVID-19 pandemic	-0.04 (-0.1, -0.03) ***	-0.1 (-0.1, -0.03) *	-0.1 (-0.1, -0.1) **	-0.01 (-0.03, 0.002)
	Slope change during COVID-19 pandemic	-0.3 (-0.4, -0.2) **	-1.0 (-1.3, -0.8) ***	-0.4 (-0.7, -0.2)	-0.7 (-0.9, -0.5) *

Abbreviations: SPC, specialized palliative care; CI, confidence interval.

Footnotes:

[a] *Level change* represents an immediate effect of the COVID-19 pandemic (the interruption on 16-Mar-2020) on home death (outcome) in the first month of the pandemic. *Trend before COVID-19 pandemic* represents an average percentage change in home death per month before the start of the COVID-19 pandemic. *Slope change during COVID-19 pandemic* represents an average percentage change in home death per month during the COVID-19 pandemic relative to the segment preceding the pandemic.

[b] All patients includes material deprivation quintiles Q1 to Q5.

* $P<0.05$

** $P<0.01$

*** $P<0.001$

© 2024 Iqbal J et al. *JAMA Network Open.*

eTable 7. Changes in Monthly Trends of Study Outcomes at Start of, Before, and During the COVID-19 Pandemic, Sensitivity Analysis 2, Excluding Patients Who Tested Positive for COVID-19 in the Last 30 Days of Life

Outcome	Parameter [a]	Monthly Trend, % per month (95% CI)			
		All Patients [b]	Least Deprived Quintile (Q1)	Intermediate Deprived Quintile (Q3)	Most Deprived Quintile (Q5)
Home death	Level change at start of COVID-19 pandemic	8.2 (7.2, 9.1) ***	11.6 (9.8, 13.5) ***	10.1 (8.9, 11.3) ***	5.7 (3.9, 7.5) **
	Trend before COVID-19 pandemic	0.02 (0.01, 0.03)	0.01 (-0.01, 0.02)	-0.01 (-0.02, 0.0004)	0.1 (0.04, 0.1) *
	Slope change during COVID-19 pandemic	-0.2 (-0.3, -0.1)	-0.7 (-0.9, -0.4) **	-0.4 (-0.6, -0.3) *	-0.02 (-0.2, 0.2)
Home death, Received SPC at end-of-life	Level change at start of COVID-19 pandemic	7.1 (5.8, 8.3) ***	7.9 (5.8, 10.0) **	8.9 (7.2, 10.6) ***	0.6 (-1.8, 2.9)
	Trend before COVID-19 pandemic	0.01 (-0.002, 0.03)	-0.01 (-0.03, 0.02)	-0.01 (-0.03, 0.002)	0.1 (0.02, 0.1) *
	Slope change during COVID-19 pandemic	-0.2 (-0.4, -0.1)	-0.3 (-0.6, -0.1)	-0.4 (-0.6, -0.2)	0.5 (0.2, 0.8)
Home death, No SPC at end-of-life	Level change at start of COVID-19 pandemic	12. 5 (11.4, 13.5) ***	19.3 (17.2, 21.3) ***	13.8 (11.7, 15.9) ***	14.5 (12.9, 16.2) ***
	Trend before COVID-19 pandemic	-0.04 (-0.1, -0.03) **	-0.1 (-0.1, -0.04) *	-0.1 (-0.1, -0.1) **	-0.02 (-0.03, -0.001)
	Slope change during COVID-19 pandemic	-0.3 (-0.4, -0.2) *	-1.1 (-1.3, -0.8) ***	-0.4 (-0.7, -0.2)	-0.6 (-0.8, -0.4) **

Abbreviations: SPC, specialized palliative care; CI, confidence interval.
Footnotes:
[a] *Level change* represents an immediate effect of the COVID-19 pandemic (the interruption on 16-Mar-2020) on home death (outcome) in the first month of the pandemic. *Trend before COVID-19 pandemic* represents an average percentage change in home death per month before the start of the COVID-19 pandemic. *Slope change during COVID-19 pandemic* represents an average percentage change in home death per month during the COVID-19 pandemic relative to the segment preceding the pandemic.
[b] All patients includes material deprivation quintiles Q1 to Q5.
[*] $P<0.05$
[**] $P<0.01$
[***] $P<0.001$

© 2024 Iqbal J et al. *JAMA Network Open.*

eTable 8. Changes in Monthly Trends of Study Outcomes at Start of, Before, and During the COVID-19 Pandemic, Sensitivity Analysis 3, Excluding Patients Who Died With Stage I and II Cancers

Outcome	Parameter [a]	Monthly Trend, % per month (95% CI)			
		Overall [b]	Q1	Q3	Q5
Home death	Level change at start of COVID-19 pandemic	8.2 (7.3, 9.1) ***	13.0 (11.5, 14.6) ***	9.7 (8.4, 11.0) ***	4.9 (3.0, 6.9) *
	Trend before COVID-19 pandemic	0.01 (-0.001, 0.02)	-0.02 (-0.04, -0.01)	-0.02 (-0.03, -0.01)	0.04 (0.02, 0.1)
	Slope change during COVID-19 pandemic	-0.3 (-0.4, -0.2) *	-0.8 (-1.0, -0.6) **	-0.5 (-0.6, -0.3) *	-0.1 (-0.3, 0.2)
Home death, Received SPC at end-of-life	Level change at start of COVID-19 pandemic	7.0 (5.8, 8.2) ***	8.9 (7.0, 10.9) ***	9.4 (7.8, 11.0) ***	1.2 (-1.2, 3.5)
	Trend before COVID-19 pandemic	0.01 (-0.01, 0.03)	-0.03 (-0.04, 0.01)	-0.02 (-0.03, 0.02)	0.1 (0.02, 0.1)
	Slope change during COVID-19 pandemic	-0.3 (-0.4, -0.2)	-0.4 (-0.6, -0.2)	-0.5 (-0.7, -0.3) *	0.2 (-0.1, 0.5)
Home death, No SPC at end-of-life	Level change at start of COVID-19 pandemic	12.0 (10. 7, 13.2) ***	16.8 (13.4, 20.1) ***	11.7 (9.5, 13.9) ***	11.9 (9.1, 14.6) ***
	Trend before COVID-19 pandemic	-0.04 (-0.1, -0.03) **	-0.1 (-0.1, -0.03)	-0.1 (-0.1, -0.1) **	-0.02 (-0.1, 0.01)
	Slope change during COVID-19 pandemic	-0.3 (-0.5, -0.2) *	-0.8 (-1.1, -0.4)	-0.3 (-0.6, -0.1)	-0.4 (-0.7, -0.1)

Abbreviations: SPC, specialized palliative care; CI, confidence interval.

Footnotes:

[a] *Level change* represents an immediate effect of the COVID-19 pandemic (the interruption on 16-Mar-2020) on home death (outcome) in the first month of the pandemic. *Trend before COVID-19 pandemic* represents an average percentage change in home death per month before the start of the COVID-19 pandemic. *Slope change during COVID-19 pandemic* represents an average percentage change in home death per month during the COVID-19 pandemic relative to the segment preceding the pandemic.

[b] All patients includes material deprivation quintiles Q1 to Q5.

* $P<0.05$

** $P<0.01$

*** $P<0.001$

© 2024 Iqbal J et al. *JAMA Network Open.*

eFigure 1. Study Cohort

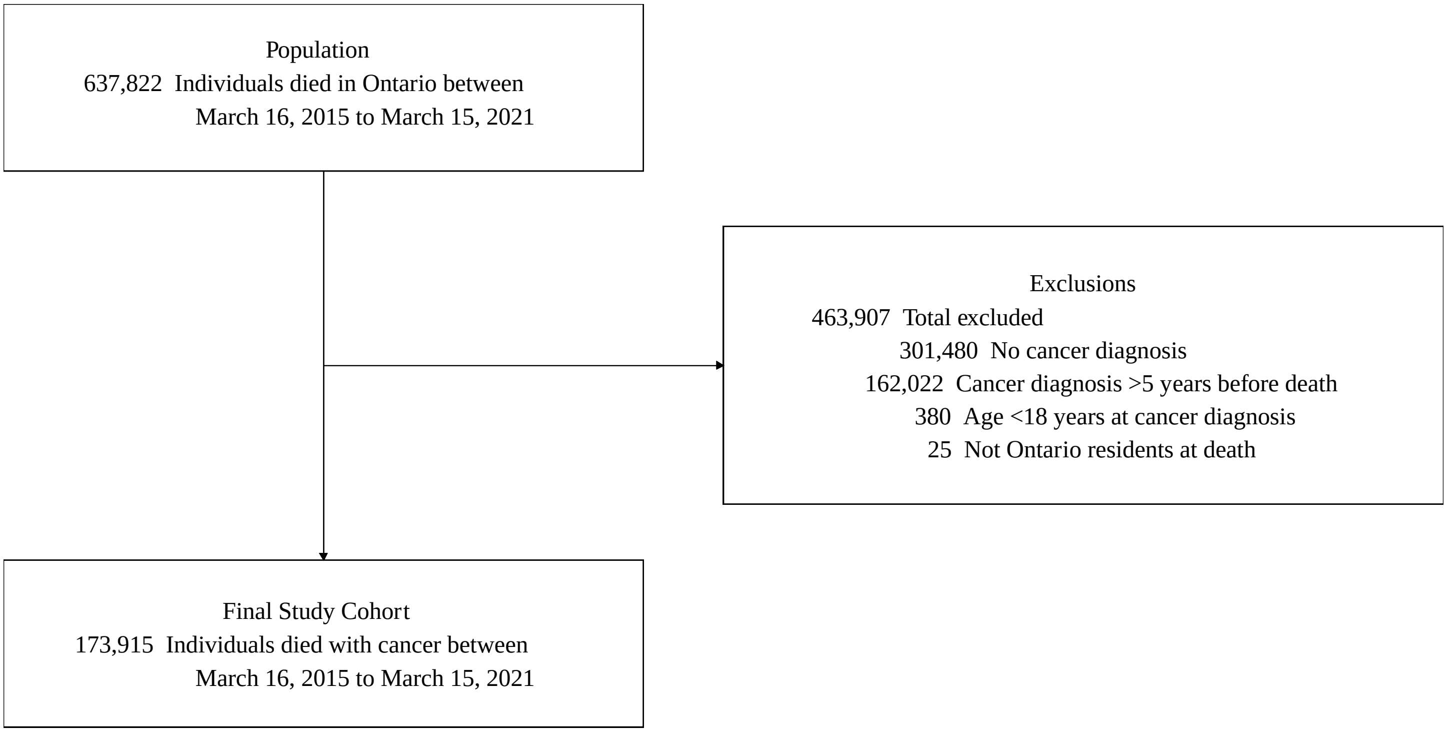

© 2024 Iqbal J et al. *JAMA Network Open.*

eFigure 2. Absolute Number of Monthly Deaths, March 16, 2015, to March 15, 2021

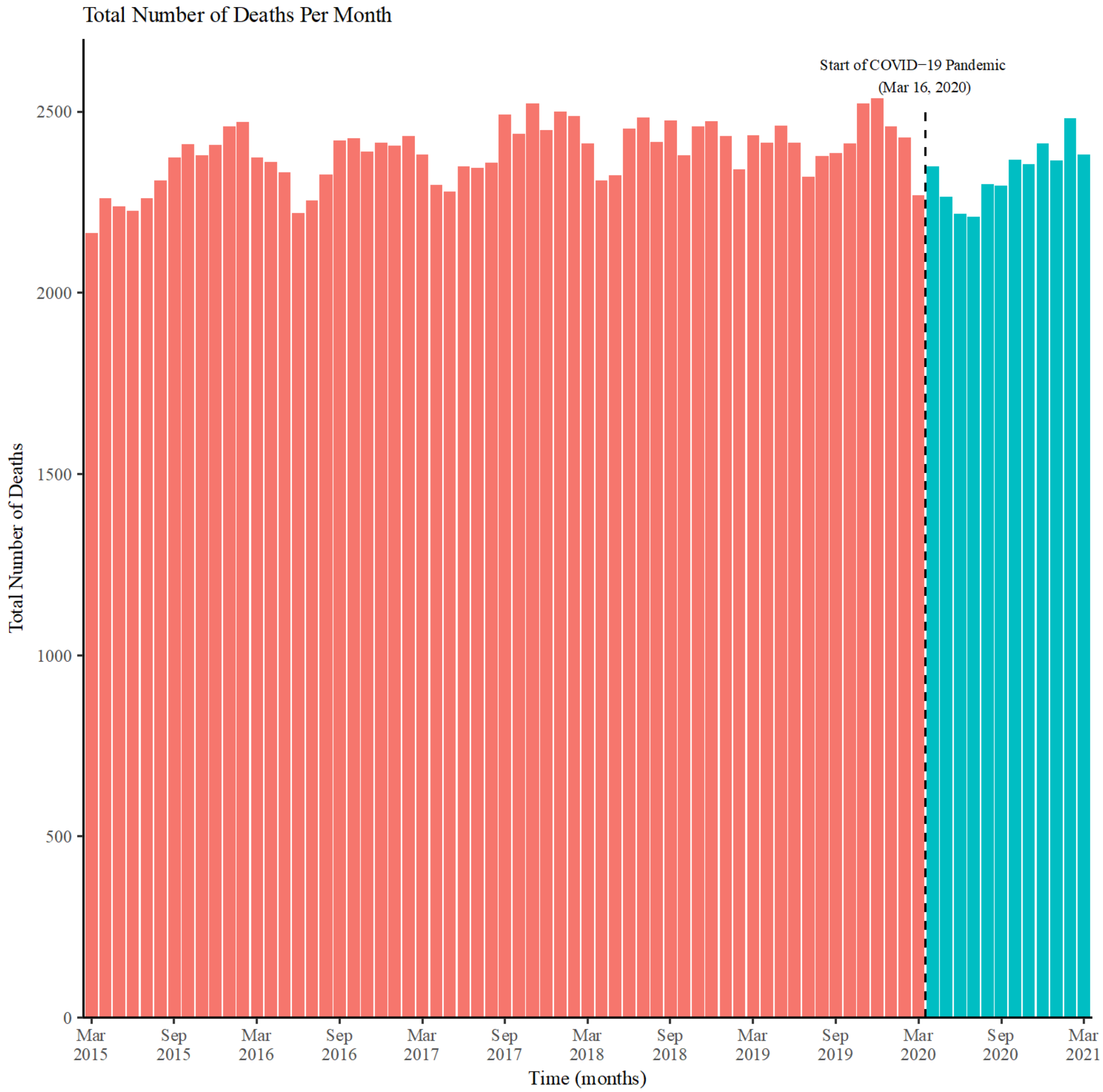

© 2024 Iqbal J et al. *JAMA Network Open.*
